# Identification of inflammatory and vascular markers associated with mild cognitive impairment

**DOI:** 10.18632/aging.101924

**Published:** 2019-04-30

**Authors:** Xue-Ning Shen, Yanxia Lu, Crystal Tze Ying Tan, Ling-Yun Liu, Jin-Tai Yu, Lei Feng, Anis Larbi

**Affiliations:** 1 Department of Neurology, Institute of Neurology, Huashan Hospital, Shanghai Medical College, Fudan University, Shanghai, China; 2 Singapore Immunology Network (SIgN), Agency for Science, Technology and Research, Singapore, Singapore; 3 Department of Psychological Medicine, Yong Loo Lin School of Medicine, National University of Singapore, Singapore, Singapore; 4 Department of Neurology, Yangpu Hospital, Tongji University School of Medicine, Shanghai, China; 5 Department of Microbiology and Immunology, Yong Loo Lin School of Medicine, National University of Singapore, Singapore, Singapore; 6 Department of Biology, Faculty of Sciences, University Tunis El Manar, Tunis, Tunisia

**Keywords:** mild cognitive impairment, TNF-α, C-peptide, VEGF-A, PAI-1, sTNFR-1, sIL-2Rα

## Abstract

Biochemical processes have been associated with the pathogenesis of mild cognitive impairment (MCI) and dementia, including chronic inflammation, dysregulation of membrane lipids and disruption of neurotransmitter pathways. However, research investigating biomarkers of these processes in MCI remained sparse and inconsistent. To collect fresh evidence, we evaluated the performance of several potential markers in a cohort of 57 MCI patients and 57 cognitively healthy controls. MCI patients showed obviously increased levels of plasma TNF-α (p = 0.045) and C-peptide (p = 0.004) as well as decreased levels of VEGF-A (p = 0.042) and PAI-1 (p = 0.019), compared with controls. In addition, our study detected significant correlations of plasma sTNFR-1 (MCI + Control: B = -6.529, p = 0.020; MCI: B = -9.865, p = 0.011) and sIL-2Rα (MCI + Control: B = -7.010, p = 0.007; MCI: B = -11.834, p = 0.003) levels with MoCA scores in the whole cohort and the MCI group. These findings corroborate the inflammatory and vascular hypothesis for dementia. Future studies are warranted to determine their potential as early biomarkers for cognitive deficits and explore the related mechanisms.

## Introduction

Dementia prominently threatens a growing number of the aging population and much effort has been devoted to early diagnosis and prediction of dementing disorders. Mild cognitive impairment (MCI) is a clinical syndrome presenting cognitive decline with preservation of global intellectual abilities and minimal interference in instrumental activities of daily living [1].

It is defined as the intermediate stage between intact cognitive function and clinically proven dementia, during which patients will benefit greatly if they obtain prompt diagnosis and efficacious intervention [1,2]. Epidemiologic studies have revealed a prevalence above 6.7% of MCI in persons aged over 60 and a growing annual rate of MCI progression to dementia [1]. Therefore, identifying MCI at an early stage and placing it in an appropriate clinical context have become an urgent and great challenge to physicians. Information of cerebrospinal fluid (CSF) and peripheral blood biomarkers as well as neuroimaging changes, such as positron emission tomography (PET) imaging of amyloid-β (Aβ) and tau, are of vital importance to facilitate clinical trials of disease-modifying therapy. The utilization of biomarker performance in detecting early dementia has shown promising diagnostic and therapeutic implications [3], and recent international guidelines have highlighted the significance of identifying putative biomarkers to distinguish MCI [2]. Blood samples, for example plasma, have more advantages over CSF and PET data in discriminating disease status, such as less invasiveness and low cost.

Many biochemical processes are involved in the pathogenesis of MCI and dementia such as Alzheimer’s disease (AD), including aberrant amyloid metabolism, phosphorylation of tau protein, dysregulation of membrane lipids and disruption of neurotransmitter pathways [4,5]. Accumulating evidence has demonstrated that multiple blood-based markers are associated with these neurodegenerative disorders, and some are closely correlated with disease progression [6-9]. Meta-analyses of AD have proved that various peripheral markers differed between AD patients and healthy controls, indicating a pivotal role of chronic inflammation in AD [10]. Furthermore, recent studies have strongly reinforced the vascular hypothesis in dementia as well as the corresponding biomarkers, such as platelet-derived growth factor receptor-β, which can act as early predictor of cognitive trajectories [11]. Since MCI patients are at risk of progressing to AD or other types of dementia, it is reasonable to speculate that those pathological alterations observed in dementia may be detected in MCI. However, current research focusing on peripheral changes of these markers in MCI have been sparse and provided inconsistent results. Few studies have systematically investigated the synergistic role of a wide array of plasma biomarkers in MCI.

The objectives of this study were to compare plasma-based marker performance between patients with MCI and cognitively healthy controls in Singapore, and to investigate whether these markers are associated with cognitive dysfunction. Based on relevant literature, meta-analyses and our team’s previous findings, 21 potentially reliable markers, which is involved in the neuroinflammation, metabolic and vascular mechanisms of MCI and dementia, were selected for evaluation owing to their previous reports on positive results [12].

## RESULTS

### Demographic and clinical characteristics

A total of 114 subjects (57 MCI patients and 57 normal controls) were ultimately enrolled in this study. Table 1 shows the clinical information and neuropsychological performance of the MCI and control groups. Matched for gender, MCI patients were relatively older (p < 0.001) and less educated (p = 0.004) than normal controls. No significant differences were noted in the frequency of diabetes mellitus, alcohol intake, smoking, marriage and employment status between MCI patients and normal controls (p > 0.05). Compared to normal controls, MCI patients performed significantly poorer on Singapore-Modified Mini-Mental State Exam (SM-MMSE) (p < 0.001) and Montreal Cognitive Assessment (MoCA) (p < 0.001), with notably higher scores of Geriatric Depression Scale (GDS) (p < 0.001) and Geriatric Anxiety Inventory (GAI) (p = 0.001).

**Table 1:** Demographic characteristics and neuropsychological performance.

	MCI	Control	p Value
N	57	57	-
Age (years)	68.77 ± 5.47	67.77 ± 5.16	< 0.001
Gender (male/female)	39/18	39/18	-
Education (years)	4.21 ± 4.84	6.26 ± 3.92	0.004
Smoking (n, %)	5 (8.8%)	3 (5.3%)	0.714
Alcohol (n, %)	11 (19.3%)	5 (8.8%)	0.106
Diabetes Mellitus (n, %)	13 (22.8%)	7 (12.3%)	0.140
Marriage (married/single)	42/15	39/18	0.536
Employment (employed, %)	8 (14.0%)	9 (15.8%)	0.793
Neuropsychological Performance			
MMSE score	25.82 ± 2.44	29.46 ± 0.66	< 0.001
MoCA score	22.93 ± 3.81	27.37 ± 2.30	< 0.001
GDS score	1.68 ± 2.16	0.54 ± 0.73	< 0.001
GAI score	1.00 ± 2.10	0.16 ± 0.62	0.001

Note: Continuous variables are expressed by mean ± SD. Comparisons between groups were analyzed as described in the Methods. Groups: MCI = patients with mild cognitive impairment; Control = cognitively healthy controls. Abbreviations: N, number of subjects in each group; MMSE, Mini-Mental State Exam; MoCA, Montreal Cognitive Assessment; GDS, Geriatric Depression Scale; GAI, Geriatric Anxiety Inventory.

## Performance of plasma markers across diagnostic groups

Table 2 summarizes the plasma concentration of all 21 analytes and illustrates the results of comparisons between groups. TNF-α (p = 0.045) and C-peptide (p = 0.004) were found significantly elevated in MCI patients compared with controls, while decreases in VEGF-A (p = 0.042) and PAI-1 (p = 0.019) were observed. No significant differences were found in comparisons of other plasma markers between MCI patients and normal controls (p > 0.05).

**Table 2:** Plasma marker levels in MCI patients and controls.

Markers		MCI			Control			p Value
		Concentration	n		Concentration	n		
TNF-α (pg/ml)		15.43 ± 2.92	48		14.29 ± 2.54	47		**0.045**
sTNFR-1 (pg/ml)		6585.75 ± 2053.00	56		6096.99 ± 1717.44	54		0.179
sTNFR-2 (pg/ml)		2196.74 ± 489.14	23		2146.16 ± 406.87	27		0.442
hsIL-6 (pg/ml)		0.77 ± 0.53	51		0.90 ± 0.70	41		0.514
sgp130 (pg/ml)		153537.29 ± 16682.29	55		147593.26 ± 25570.74	53		0.335
IP-10 (pg/ml)		84.41 ± 67.36	56		63.66 ± 33.20	54		0.089
CXCL13 (pg/ml)		107.71 ± 37.63	56		109.34 ± 42.30	54		0.853
sIL-2Rα (pg/ml)		763.96 ± 199.31	56		703.31 ± 228.54	54		0.140
6Ckine (pg/ml)		225.59 ± 41.04	56		216.78 ± 58.42	54		0.361
CTACK (pg/ml)		530.80 ± 191.41	56		496.17 ± 194.54	54		0.349
hsCRP (μg/ml)		1.46 ± 2.54	55		1.49 ± 2.38	53		0.513
IL-8 (pg/ml)		5.98 ± 3.38	53		7.70 ± 7.20	49		0.212
C-peptide (pg/ml)		917.38 ± 549.46	55		704.04 ± 639.51	52		**0.004**
MCP-1 (pg/ml)		214.10 ± 62.84	55		242.43 ± 119.57	52		0.535
VEGFA (pg/ml)		41.55 ± 24.43	55		56.49 ± 37.95	53		**0.042**
IL-4 (pg/ml)		22.66 ± 7.59	43		27.38 ± 15.22	45		0.277
Leptin (pg/ml)		9437.62 ± 8363.95	51		9020.54 ± 6211.62	47		0.698
PAI-1 (pg/ml)		45697.87 ± 29009.55	53		62957.59 ± 40050.37	51		**0.019**
NTproBNP (pg/ml)		1217.56 ± 730.01	42		1461.21 ± 819.73	35		0.141
TIMP-2 (pg/ml)		38369.49 ± 13863.34	48		37787.49 ± 16234.35	47		0.639
T4 Total (nmol/L)		105.23 ± 23.35	52		101.27 ± 28.61	41		0.371

Note: Continuous variables are expressed by mean ± SD. Comparisons between groups were analyzed as described in the Methods. Significance (bold values), p < 0.05. Groups: MCI = patients with mild cognitive impairment; Control = cognitively healthy controls.

### Correlations of cognitive performance with age and education

The potential correlations of clinical characteristics with cognition level assessed by SM-MMSE and MoCA scores were evaluated by Spearman analysis. Age was found obviously inversely correlated with MoCA score in MCI (ρ = -0.275, p = 0.041) and control (ρ = -0.278, p = 0.041) subgroups as well as the whole sample (ρ = -0.286, p = 0.002), but with SM-MMSE score only in the MCI subgroup (ρ = -0.339, p = 0.010). Years of education were noted significantly related to SM-MMSE (Total: ρ = 0.523; MCI: ρ = 0.530; CN: 0.601; all p < 0.001) and MoCA (Total: ρ = 0.447; MCI: ρ = 0.391; CN: 0.392; all p < 0.005) scores in all the analyses whether performed separately in MCI and control groups or performed among the whole sample.

### Correlations of plasma marker levels with cognitive performances

**Figure 1:**
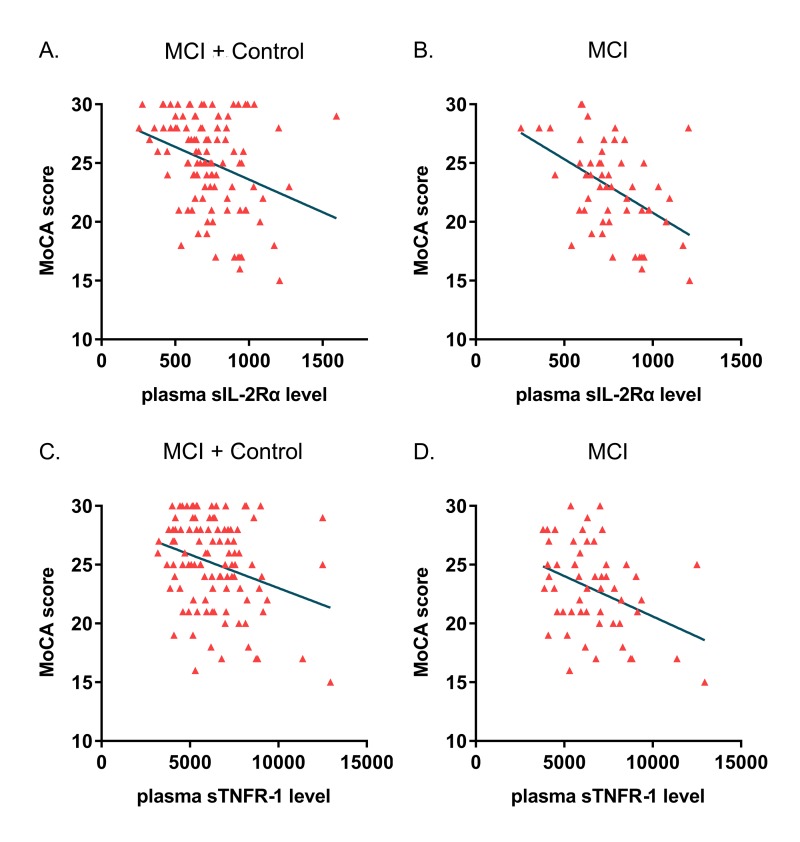
**Correlations of plasma sIL-2Rα and sTNFR-1 level with cognitive impairment.** Scatter plots illustrated the correlations of plasma sIL-2Rα and sTNFR-1 concentration with cognitive level evaluated by MoCA scores. (**A**) The correlation of sIL-2Rα with MoCA in the whole cohort (MCI + Control: ρ = -0.305, p = 0.001). (**B**) The correlation of sIL-2Rα with MoCA in MCI patients (ρ = -0.464, p < 0.001). (**C**) The correlation of sTNFR-1 with MoCA in the whole cohort (MCI + Control: ρ = -0.235, p = 0.015). (**D**) the correlation of sTNFR-1 with MoCA in MCI patients (ρ = -0.314, p = 0.019).

As listed in the Supplementary Table 1, the potential correlations of these 21 plasma markers with cognitive performances were analyzed in the whole cohort, MCI group and control group (in the unadjusted and adjusted models). The Spearman correlation analysis implied that the SM-MMSE and MoCA scores were inversely correlated with plasma sIL-2Rα concentration in both the whole cohort (SM-MMSE: ρ = -0.207, p = 0.030; MoCA: ρ = -0.305, p = 0.001) and MCI group (SM-MMSE: ρ = -0.269, p = 0.045; MoCA: ρ = -0.464, p < 0.001) (Figure 1). In addition, MoCA scores were inversely associated with plasma sTNFR-1 levels in the whole cohort (ρ = -0.235, p = 0.015) and MCI group (ρ = -0.314, p = 0.019) (Figure 1). Furthermore, we also detected positive correlations of IL-8 (ρ = 0.298, p = 0.042) and C peptide (ρ = 0.282, p = 0.047) with the MoCA scores in the control group. In partial correlation analyses adjusting for age, gender, education, GDS and GAI scores, inverse correlations were demonstrated of SM-MMSE (r = -0.198, p = 0.045) and MoCA (r = -0.230, p = 0.020) scores with sTNFR-1 level and of only MoCA (r = -0.244, p = 0.013) score with sIL-2Rα level in the whole cohort. In MCI subgroup, the MoCA scores were found inversely linked to the plasma concentration of sIL-2Rα (r = -0.420, p = 0.002) and sTNFR-1 (r = -0.357, p = 0.010). Plasma 6Ckine levels presented a potential link with SM-MMSE score (r = -0.282, p = 0.045) in the MCI patients. In normal controls, MoCA scores were positively correlated with C-peptide level (r = 0.389, p = 0.008) and negatively with NTproBNP level (r = -0.481, p = 0.008), while no significant correlation of these two markers was found in the MCI group.

### Linear regression analysis and ROC curves

We first used the simple linear regression model to select the potentially independent variables (Supplementary Table 2). Then, the multivariable linear regression analysis was utilized to clarify those independent variables that were correlated with the prevalence of cognitive impairment in the study cohort. SM-MMSE and MoCA scores were set as dependent variables, while plasma marker concentration, age, gender, education, GDS and GAI scores were considered as independent variables. Results indicated that MoCA score was significantly associated with plasma sIL-2Rα and sTNFR-1 in both the whole cohort and MCI patients (p < 0.05) (Table 3). In addition, MoCA was also found correlated with plasma C-peptide in normal controls (B = 2.111, p = 0.018) (Table 4). The power of each linear regression test was calculated. Among them, sTNFR-1 (> 70%) and sIL-2Rα (> 90%) showed relatively reliable test performances, while that of C-peptide was 53.9%. The calculated power for other markers varied. To further evaluate the performance of these markers in predicting disease status, we conducted the receiver operating characteristic (ROC) analyses in the study cohort. Statistics of areas under the curves (AUCs) showed acceptable predictive abilities of these plasma-based markers in distinguishing MCI from normal controls (VEGF-A AUC = 0.614, p = 0.042; PAI-1 AUC = 0.633, p = 0.019; TNF-α AUC = 0.615, p = 0.054; C peptide AUC = 0.662, p = 0.004).

**Table 3:** Assessment results of the risk of having cognitive impairment in the multiple linear regression model across different groups (sIL-2Rα and sTNFR-1).

Markers			MCI + Control				MCI		
			B	SE	p value		B	SE	p value
sIL-2Rα			-7.010	2.546	**0.007**		-11.834	3.774	**0.003**
	Age		-0.080	0.067	0.239		-0.070	0.092	0.452
	Gender		-0.206	0.773	0.790		-0.905	1.086	0.409
	Education		0.277	0.083	**0.001**		0.180	0.113	0.117
	GDS		-0.181	0.238	0.450		0.077	0.258	0.766
	GAI		-0.096	0.253	0.704		-0.044	0.264	0.869
sTNFR-1			-6.529	2.758	**0.020**		-9.865	3.708	**0.011**
	Age		-0.075	0.068	0.274		-0.065	0.095	0.496
	Gender		-0.287	0.783	0.714		-0.817	1.112	0.466
	Education		0.303	0.083	**0.000**		0.259	0.113	**0.027**
	GDS		-0.191	0.241	0.428		0.098	0.264	0.712
	GAI		-0.070	0.256	0.786		-0.019	0.272	0.943

Note: Dependent variable: MoCA scores; B, regression coefficients; SE, standard error. Groups: MCI = patients with mild cognitive impairment; Control = cognitively healthy controls. Significance (bold values), p < 0.05.

**Table 4:** Assessment results of the risk of having cognitive impairment in the multiple linear regression model across different groups (C-peptide).

Markers			MCI + Control				Control		
			B	SE	p value		B	SE	p value
C-peptide			-0.999	1.038	0.338		2.111	0.858	**0.018**
	Age		-0.113	0.070	0.107		-0.120	0.061	0.056
	Gender		-0.177	0.826	0.830		-0.076	0.714	0.916
	Education		0.292	0.087	**0.001**		0.162	0.081	0.052
	GDS		-0.174	0.248	0.483		0.205	0.438	0.642
	GAI		-0.109	0.263	0.679		0.432	0.499	0.391

Note: Dependent variable: MoCA scores; B, regression coefficients; SE, standard error. Groups: MCI = patients with mild cognitive impairment; Control = cognitively healthy controls. Significance (bold values), p < 0.05.

## DISCUSSION

The present study provided evidence indicating that individuals with MCI differed from cognitively healthy controls in the plasma expression of at least four markers: TNF-α, VEGF-A, C-peptide and PAI-1. In addition to advanced age and low education level, plasma sIL-2Rα and sTNFR-1 concentrations were negatively associated with MoCA scores in all participants and MCI patients, implying a potential correlation of these markers with MCI. Taken together, our findings have further corroborated the inflammatory responses and meta-vascular changes in the pathophysiological cascade of MCI.

### Regulation of inflammation

Neuroinflammation has been well documented to play a role in the early phase of neurodegeneration [13-15]. In this cross-sectional study, we investigated the plasma performance of several inflammatory markers in MCI and control groups, including cytokine hsIL-6 and one of its receptors sgp130, chemokine IL-8, and inflammation associated protein VEGF-A.

Among these results, plasma TNF-α levels proved to be mildly but significantly elevated in MCI patients compared with normal controls. As an important mediator that could activate central innate immune responses, TNF-α has been associated with the dysfunction and death of neurons [16]. It is widely accepted that TNF-α exerts effects by inducing Aβ production in vitro and mediating toxicity induced by amyloidosis [17-19]. Some researchers reported that the pro-inflammatory marker TNF-α increased in the peripheral blood or CSF of patients suffering from Alzheimer’s disease [20,21]. In their studies, there was a trend of higher TNF-α expression in the MCI group compared to healthy controls, yet the data were not statistically significant [20]. However, some studies suggested there was no change or lower expression of TNF-α in AD and MCI patients compared to controls [22,23]. Inconclusive results may be due to different methodologies across laboratories. It could also be because that levels of TNF-α change during neurodegeneration but not in the very early stage of disease, or that the underlying alterations are too slight to be detected. In humans, TNF-α exerts its effect by binding to the transmembrane receptors: TNF receptor 1 and TNF receptor 2 (TNFR-1, TNFR-2) [24]. Soluble TNFR-1 and TNFR-2 circulate for longer than the pro-inflammatory cytokine itself, and therefore they are suitable for regulating TNF activity [25]. We detected a trend of increased plasma sTNFR-1 level in MCI patients compared with controls, but data did not show statistical significance. However, levels of plasma sTNFR-1 were demonstrated to have a significantly inverse correlation with MoCA score in both the whole cohort and the MCI group, indicating its involvement in MCI and potential capacity for detecting disease progression. Our results had no positive findings for plasma sTNFR-2, which was not in line with the result from previous research conducted by Gao et al [26], reaffirming the conclusion from previous studies that sTNFR-1 and sTNFR-2 may be activated and expressed differentially in the neurodegeneration of AD [27]. But given the small sample size and data lost for out of range or undetectable, it should be interpreted with caution.

Aging has been associated with alterations of circulating cells producing cytokines or chemokines, and changes in the distribution of lymphocyte subset might be specific in different types of neurodegenerative disorders such as AD [28]. In agreement with a previous clinical study [26], our research data showed significantly inverse correlations between plasma sIL-2Rα concentration and cognition level evaluated by MoCA in both the whole study cohort and the MCI group. But this correlation was not detected in the control group. It is known that IL-2Rα plays a critical role in the signaling of IL-2 and the regulation of immune reactions [29]. Produced from the membrane-bound IL-2Rα, the soluble IL-2Rα could inhibit the IL-2 signaling and promote the proliferation of T cell, and was therefore regarded as the stable biomarker for T cell activation [30,31]. Laurent et al. have demonstrated a pivotal role of T cell infiltration in tauopathy and cognitive deficits in neurodegeneration [32]. Altogether, it is reasonable to speculate that there is an association between plasma sIL-2Rα level and cognitive impairment, which might be utilized for detecting disease progression.

### Metabolic and vascular alterations

Metabolic and vascular mechanisms have been suggested to contribute to MCI and dementia, yet the previous studies displayed discrepant results [33,34]. We explored the potential role of aberrant metabolism and vascular function in MCI by measuring plasma levels of promising markers, including PAI-1, C-peptide and VEGF-A.

Elevation in PAI-1, a serine protease inhibitor (Serpin), also known as endothelial plasminogen activator inhibitor, confers high risk of vascular diseases such as dementia [33]. PAI-1 is considered as a primary factor in regulating the balance between thrombosis and fibrinolysis, and it has an effect on reducing brain Aβ clearance [34]. Our study found plasma PAI-1 levels decreased in MCI individuals compared to controls, which was contrary to some previous findings [33,35,36]. Oh et al. indicated elevated plasma PAI-1 levels in Korean MCI patients compared to controls, and yet a similar situation was not detected in a cohort of Austrian MCI patients studied by Marksteiner et al. [35]. On the contrary, Trollor et al. demonstrated a lower PAI-1 concentration in amnestic MCI patients compared to cognitively normal controls [37]. Since MCI is heterogeneous, following by several outcomes, and plasma PAI-1 levels could be modulated by multiple factors such as ethnicity, genetics and medication, the inconsistency was largely caused by insufficient studies and the great heterogeneity among them. More comprehensive analyses from multi-centered, large-scale researches are warranted in the future to obtain definite conclusions.

Accumulating evidence supported that C-peptide was associated with inflammation and cognitive function in middle-aged or older patients with diabetes mellitus (DM), yet there were disagreements [8,9]. Chen et al. observed decreased C-peptide level in MCI patients and positive correlation of MoCA with C-peptide in patients with type 2 diabetes mellitus (T_2_DM) [8]. In contrast, Zhou et al. reported an inverse association of SM-MMSE with fasting C-peptide but no significant difference in C-peptide level between these two groups, whether in the non-DM or DM subjects [9]. We found obviously elevated C-peptide concentration in the plasma of MCI patients compared with cognitively healthy controls. Regression analysis showed a trend of positive correlation between C-peptide level and MoCA score in the control group, but no positive findings in the whole cohort or the MCI group. Since DM duration was widely reported to be correlated with cognition function, it could explain part of the inconsistency because we did not include DM duration in the analyses [8]. Different types of DM also seem to partly account for the inconsistency, as evidenced by the reports that the absence of C-peptide in T_1_DM patients could cause damage whereas the excess in T_2_DM patients may be harmful [38,39]. Though DM patients in our cohort represent a small fraction, we could not exclude the effects of medication on modulating the levels of meta-inflammatory markers, such as Metformin. Further studies are required to address these aspects.

Dysfunction of the Blood-Brain Barrier (BBB) is a key pathogenic step in initiation and progression of inflammatory lesions in the brain [11,40]. Released by activated astrocytes, vascular endothelial growth factor A (VEGF-A) has been associated with the downregulation of endothelial tight junction transmembrane proteins, which induced the BBB breakdown [40,41]. A previous animal study illustrated that VEGF-A and its upstream mediator lipocalin-2 play a critical role in the BBB breakdown in hippocampus of the vascular dementia (VaD) [42]. The upregulation of these proteins might partly explain the neurotoxicity and neuroinflammation in VaD [42]. In contrast, our research found lower plasma levels of VEGF-A in patients with MCI compared to normal controls. This inconsistency may result from increased soluble VEGF receptors expression in MCI donors, which was not tested in our study. One study did report the reduced circulating sVEGF-R2 levels in AD patients compared to controls [43]. The inconsistency between our result and those of the previous studies may be explained by the timing and stage of the disease. It is likely that the balance between VEGF1/2 and VEGF-R1/2 (and other markers and their receptors) in healthy and MCI individuals is maintained by the regulation we observed and that the disruption of the regulation may accelerate disease onset and progression. One should also consider that the above-mentioned regulations also occur in the brain when we are testing circulating biomarkers. Future studies combining peripheral, CSF and brain tissue data are required for confirmation.

### Cognitive assessment

In a recent meta-analysis, the MoCA test has been proved to have better sensitivity and specificity than the SM-MMSE test to screen MCI from intact cognition in population aged over 60 [44]. It involves important cognitive domains and shows good reliability in repeated testing [45]. Our research data showed that the concentration of some plasma-based markers was positively or negatively correlated with MoCA scores rather than with SM-MMSE scores. It supported the notion that the MoCA test could better detect cognitive impairment than SM-MMSE, suggesting it as a highly sensitive tool for clinicians. Besides, the possibility could not be ruled out that other plasma-based markers have no correlation with cognitive impairment. Several reasons may account for this result in our study. When compared with normal controls, MCI patients present more minor variations of cognition decline than patients with dementia, which may enhance the difficulty in detecting disease progression. In the process of sampling, part of the data were excluded from analysis because of concentration undetectable or out of range. Large-scale studies containing healthy controls and patients with dementia and MCI are demanded to reach more powerful conclusions.

### Limitations

It should be noted that there were some limitations in our research. First, the relatively small sample size may restrict our ability to detect the associations of other inflammatory and metabolic markers with cognitive impairment. Second, patients with the Alzheimer’s disease or other types of dementia were not enrolled in the present study. And MCI subtypes which may progress into different types of dementia were not analyzed separately, which may reduce the strength of current evidence. Finally, longitudinal, multi-centered studies are needed to focus on the progression of MCI and alterations of plasma-based markers simultaneously.

## CONCLUSION

In summary, our data indicated elevated TNF-α and C-peptide as well as lowered VEGF-A and PAI-1 in the plasma of MCI patients, supporting the involvement of inflammatory and meta-vascular alterations in the pathogenesis of MCI. In addition, our study suggested that plasma sTNFR-1 and sIL-2Rα might be utilized as potential markers for cognitive impairment in the early stages of dementia. Future studies are required to determine whether these markers could be biomarkers for clinical diagnosis and to investigate the underlying mechanism.

## METHODS

### Ethics

This study was conducted at the National University of Singapore. All procedures were compliant with the international guidelines and ethical standards, and the study protocol was reviewed and approved by the National University of Singapore Institutional Review Board. Written informed consent was obtained from all participants or authorized representatives before entering the study.

### Participants

This case-control study was conducted from July 2011 to September 2017. Elderly people aged 60 and above were recruited from the Training and Research Academy at Jurong Point, and Jurong area of Singapore. All participants underwent interviews, laboratory examinations and detailed collections of clinical data, including age, gender, education, alcohol intake, frequency of smoking, marriage and employment status, etc. A series of neuropsychological tests, including MoCA, SM-MMSE, Geriatric Depression Scale (GDS) and Geriatric Anxiety Inventory (GAI), were administered in a certain order to assess the cognitive and mental state of the participants. These evaluations were performed by experienced neuropsychiatrists on a one-to-one basis. Both participants and neuropsychiatrists were blinded to the study design.

MCI patients were diagnosed based on the widely accepted criteria advocated by Peterson and his colleagues [46,47]. Inclusion criteria for normal controls require a SM-MMSE score ≥ 28. Subjects with the presence of dementia or other neurological diseases such as Parkinson’s disease were excluded. Besides, participants were excluded if they have any of the following medical problems: acute diabetic complications, acute cerebrovascular accident or history of cerebrovascular accident within 5 years, acute cardiovascular accident or history of cardiovascular accident within 5 years, systemic disorders such as malignancy which were not cured, severe infection, drug abuse or dependency condition and severe psychiatric disorders which were not cured.

### Measurements of plasma marker concentration

Archival plasma specimens were thawed and quantitative assays were performed for 21 analytes of cytokines, chemokines, hormones, metabolites and other biomarkers which were screened from previously published literature to be correlated with cognitive function, MCI or dementia. Luminex assays were used to measure the levels of the tumor necrosis factor alpha (TNF-α) and its soluble receptor sTNFR-1, sTNFR-2, interferon-γ-inducible protein-10 (IP-10), chemokine C-X-C motif ligand 13 (CXCL13), the soluble interleukin-2 receptor alpha (sIL-2Rα), 6Ckine (C-C motif chemokine ligand 21, CCL21), CTACK (C-C motif chemokine ligand 27, CCL27), interleukin-8 (IL-8), C-peptide, monocyte chemoattractant protein 1 (MCP-1), vascular endothelial growth factor A (VEGF-A), interleukin-4 (IL-4), leptin, plasminogen activator inhibitor-1 (PAI-1), N-terminal pro-B type natriuretic peptide (NTproBNP) and tissue inhibitor of metalloproteinases 2 (TIMP2) (R&D Systems and Merck Millipore). Samples were incubated with beads, each uniquely fluorescent and conjugated to specific antibodies against the marker of interest. Detection antibodies were then added to the complex and the fluorescence was measured using a Luminex analyzer. Total thyroxine (T4), high sensitive C-reactive protein (hsCRP), high sensitive interleukin-6 (hsIL-6) and one of its receptor soluble gp130 (sgp130, a natural antagonist of IL-6 trans-signaling) were measured via the enzyme-linked immunosorbent assay (ELISA). Plasma samples were added to wells of ready-coated plates coated with antibodies specific to the marker of interest. The bound markers were then incubated with detection antibodies. The absorbance resulting from the enzymatic reaction from the enzyme on the detection antibodies and added substrate was measured using a microplate reader. All experimental procedures were standardized across runs and laboratory personnel.

### Statistical analyses

Data are presented as the mean ± standard deviation (SD) or number (percentage) as appropriate. Statistical analysis and image processing were performed using SPSS (version 25, IBM Inc., Armonk, NY, USA) and GraphPad Prism (version 7) software. Normal or non-normal distribution of continuous variables was checked by the Kolmogorov-Smirnov test. For data that obey normal distribution, comparisons between MCI patients and normal controls were conducted by the independent sample *t*-test. For non-normally distributed data, comparisons were performed using the non-parametric Wilcoxon signed rank test. Group comparisons of categorical data, including gender, alcohol intake, frequency of smoking, marriage and employment status, were analyzed using the chi-squared test. The Spearman correlation test and partial correlation analysis were used to explore the variables that might be related to cognitive impairment. Simple and multiple linear regression analysis were then conducted to investigate the possible relationship of plasma marker levels with cognition. In the regression analyses, the log_10_ transformation of plasma marker concentration was utilized to provide normally distributed data set. The performance of markers to distinguish MCI patients from normal controls was illustrated by the receiver operator characteristic curve (ROC). Considering the small sample size, we calculated the power of the test using the PASS15 software. A two-tailed p-value of < 0.05 was regarded as statistically significant.

## Supplementary Material

Supplementary Tables

## References

[1] Petersen RC, Lopez O, Armstrong MJ, Getchius TS, Ganguli M, Gloss D, Gronseth GS, Marson D, Pringsheim T, Day GS, Sager M, Stevens J, Rae-Grant A. Practice guideline update summary: Mild cognitive impairment: Report of the Guideline Development, Dissemination, and Implementation Subcommittee of the American Academy of Neurology. Neurology. 2018; 90:126–35. 10.1212/WNL.0000000000004826 29282327 PMC5772157

[2] Petersen RC, Caracciolo B, Brayne C, Gauthier S, Jelic V, Fratiglioni L. Mild cognitive impairment: a concept in evolution. J Intern Med. 2014; 275:214–28. 10.1111/joim.12190 24605806 PMC3967548

[3] Nakamura A, Kaneko N, Villemagne VL, Kato T, Doecke J, Doré V, Fowler C, Li QX, Martins R, Rowe C, Tomita T, Matsuzaki K, Ishii K, et al. High performance plasma amyloid-β biomarkers for Alzheimer’s disease. Nature. 2018; 554:249–54. 10.1038/nature25456 29420472

[4] de la Monte SM, Tong M. Brain metabolic dysfunction at the core of Alzheimer’s disease. Biochem Pharmacol. 2014; 88:548–59. 10.1016/j.bcp.2013.12.012 24380887 PMC4550323

[5] Procaccini C, Santopaolo M, Faicchia D, Colamatteo A, Formisano L, de Candia P, Galgani M, De Rosa V, Matarese G. Role of metabolism in neurodegenerative disorders. Metabolism. 2016; 65:1376–90. 10.1016/j.metabol.2016.05.018 27506744

[6] Buchhave P, Zetterberg H, Blennow K, Minthon L, Janciauskiene S, Hansson O. Soluble TNF receptors are associated with Aβ metabolism and conversion to dementia in subjects with mild cognitive impairment. Neurobiol Aging. 2010; 31:1877–84. 10.1016/j.neurobiolaging.2008.10.012 19070941

[7] Tan ZS, Beiser AS, Vasan RS, Roubenoff R, Dinarello CA, Harris TB, Benjamin EJ, Au R, Kiel DP, Wolf PA, Seshadri S. Inflammatory markers and the risk of Alzheimer disease: the Framingham Study. Neurology. 2007; 68:1902–08. 10.1212/01.wnl.0000263217.36439.da 17536046

[8] Chen RH, Jiang XZ, Zhao XH, Qin YL, Gu Z, Gu PL, Zhou B, Zhu ZH, Xu LY, Zou YF. Risk factors of mild cognitive impairment in middle aged patients with type 2 diabetes: a cross-section study. Ann Endocrinol (Paris). 2012; 73:208–12. 10.1016/j.ando.2012.04.009 22704263

[9] Zhou Y, Fang R, Liu LH, Chen SD, Tang HD. Clinical Characteristics for the Relationship between Type-2 Diabetes Mellitus and Cognitive Impairment: A Cross-Sectional Study. Aging Dis. 2015; 6:236–44. 10.14336/AD.2014.1004 26236545 PMC4509472

[10] Lai KS, Liu CS, Rau A, Lanctôt KL, Köhler CA, Pakosh M, Carvalho AF, Herrmann N. Peripheral inflammatory markers in Alzheimer’s disease: a systematic review and meta-analysis of 175 studies. J Neurol Neurosurg Psychiatry. 2017; 88:876–82. 10.1136/jnnp-2017-316201 28794151

[11] Nation DA, Sweeney MD, Montagne A, Sagare AP, D’Orazio LM, Pachicano M, Sepehrband F, Nelson AR, Buennagel DP, Harrington MG, Benzinger TL, Fagan AM, Ringman JM, et al. Blood-brain barrier breakdown is an early biomarker of human cognitive dysfunction. Nat Med. 2019; 25:270–76. 10.1038/s41591-018-0297-y 30643288 PMC6367058

[12] Shen XN, Niu LD, Wang YJ, Cao XP, Liu Q, Tan L, Zhang C, Yu JT. Inflammatory markers in Alzheimer’s disease and mild cognitive impairment: a meta-analysis and systematic review of 170 studies. J Neurol Neurosurg Psychiatry. 2019; 90:590–98. 10.1136/jnnp-2018-319148 30630955

[13] Ott BR, Jones RN, Daiello LA, de la Monte SM, Stopa EG, Johanson CE, Denby C, Grammas P. Blood-Cerebrospinal Fluid Barrier Gradients in Mild Cognitive Impairment and Alzheimer’s Disease: Relationship to Inflammatory Cytokines and Chemokines. Front Aging Neurosci. 2018; 10:245. 10.3389/fnagi.2018.00245 30186149 PMC6110816

[14] Heneka MT, Carson MJ, El Khoury J, Landreth GE, Brosseron F, Feinstein DL, Jacobs AH, Wyss-Coray T, Vitorica J, Ransohoff RM, Herrup K, Frautschy SA, Finsen B, et al. Neuroinflammation in Alzheimer’s disease. Lancet Neurol. 2015; 14:388–405. 10.1016/S1474-4422(15)70016-5 25792098 PMC5909703

[15] Reale M, Brenner T, Greig NH, Inestrosa N, Paleacu D. Neuroinflammation, AD, and Dementia. Int J Alzheimers Dis. 2010; 2010:2010. 10.4061/2010/974026 20871836 PMC2943141

[16] Decourt B, Lahiri DK, Sabbagh MN. Targeting Tumor Necrosis Factor Alpha for Alzheimer’s Disease. Curr Alzheimer Res. 2017; 14:412–25. 10.2174/1567205013666160930110551 27697064 PMC5328927

[17] Yamamoto M, Kiyota T, Horiba M, Buescher JL, Walsh SM, Gendelman HE, Ikezu T. Interferon-gamma and tumor necrosis factor-alpha regulate amyloid-beta plaque deposition and beta-secretase expression in Swedish mutant APP transgenic mice. Am J Pathol. 2007; 170:680–92. 10.2353/ajpath.2007.060378 17255335 PMC1851864

[18] Li R, Yang L, Lindholm K, Konishi Y, Yue X, Hampel H, Zhang D, Shen Y. Tumor necrosis factor death receptor signaling cascade is required for amyloid-beta protein-induced neuron death. J Neurosci. 2004; 24:1760–71. 10.1523/JNEUROSCI.4580-03.2004 14973251 PMC6730458

[19] Medeiros R, Prediger RD, Passos GF, Pandolfo P, Duarte FS, Franco JL, Dafre AL, Di Giunta G, Figueiredo CP, Takahashi RN, Campos MM, Calixto JB. Connecting TNF-alpha signaling pathways to iNOS expression in a mouse model of Alzheimer’s disease: relevance for the behavioral and synaptic deficits induced by amyloid beta protein. J Neurosci. 2007; 27:5394–404. 10.1523/JNEUROSCI.5047-06.2007 17507561 PMC6672347

[20] Gezen-Ak D, Dursun E, Hanagasi H, Bilgic B, Lohman E, Araz OS, Atasoy IL, Alaylioglu M, Onal B, Gurvit H, Yilmazer S. BDNF, TNFalpha, HSP90, CFH, and IL-10 serum levels in patients with early or late onset Alzheimer’s disease or mild cognitive impairment. J Alzheimers Dis.. 2013; 37:185–95. 10.3233/JAD-130497 23948885

[21] Kim YS, Lee KJ, Kim H. Serum tumour necrosis factor-α and interleukin-6 levels in Alzheimer’s disease and mild cognitive impairment. Psychogeriatrics. 2017; 17:224–30. 10.1111/psyg.12218 28130814

[22] Kim SM, Song J, Kim S, Han C, Park MH, Koh Y, Jo SA, Kim YY. Identification of peripheral inflammatory markers between normal control and Alzheimer’s disease. BMC Neurol. 2011; 11:51. 10.1186/1471-2377-11-51 21569380 PMC3120663

[23] Marksteiner J, Kemmler G, Weiss EM, Knaus G, Ullrich C, Mechtcheriakov S, Oberbauer H, Auffinger S, Hinterhölzl J, Hinterhuber H, Humpel C. Five out of 16 plasma signaling proteins are enhanced in plasma of patients with mild cognitive impairment and Alzheimer’s disease. Neurobiol Aging. 2011; 32:539–40. 10.1016/j.neurobiolaging.2009.03.011 19395124 PMC4311051

[24] Wajant H, Pfizenmaier K, Scheurich P. Tumor necrosis factor signaling. Cell Death Differ. 2003; 10:45–65. 10.1038/sj.cdd.4401189 12655295

[25] Bai L, Song N, Yu J, Tan L, Shen Y, Xie J, Jiang H. Elevated plasma levels of soluble TNFRs and TACE activity in Alzheimer’s disease patients of Northern Han Chinese descent. Curr Alzheimer Res. 2013; 10:57–62. 23368432

[26] Gao Q, Camous X, Lu YX, Lim ML, Larbi A, Ng TP. Novel inflammatory markers associated with cognitive performance: Singapore Longitudinal Ageing Studies. Neurobiol Aging. 2016; 39:140–46. 10.1016/j.neurobiolaging.2015.12.002 26923410

[27] Cheng X, Yang L, He P, Li R, Shen Y. Differential activation of tumor necrosis factor receptors distinguishes between brains from Alzheimer’s disease and non-demented patients. J Alzheimers Dis. 2010; 19:621–30. 10.3233/JAD-2010-1253 20110607 PMC3746510

[28] Speciale L, Calabrese E, Saresella M, Tinelli C, Mariani C, Sanvito L, Longhi R, Ferrante P. Lymphocyte subset patterns and cytokine production in Alzheimer’s disease patients. Neurobiol Aging. 2007; 28:1163–69. 10.1016/j.neurobiolaging.2006.05.020 16814429

[29] Malek TR, Bayer AL. Tolerance, not immunity, crucially depends on IL-2. Nat Rev Immunol. 2004; 4:665–74. 10.1038/nri1435 15343366

[30] Maier LM, Anderson DE, Severson CA, Baecher-Allan C, Healy B, Liu DV, Wittrup KD, De Jager PL, Hafler DA. Soluble IL-2RA levels in multiple sclerosis subjects and the effect of soluble IL-2RA on immune responses. J Immunol. 2009; 182:1541–47. 10.4049/jimmunol.182.3.1541 19155502 PMC3992946

[31] Maier LM, Lowe CE, Cooper J, Downes K, Anderson DE, Severson C, Clark PM, Healy B, Walker N, Aubin C, Oksenberg JR, Hauser SL, Compston A, et al, and International Multiple Sclerosis Genetics Consortium. IL2RA genetic heterogeneity in multiple sclerosis and type 1 diabetes susceptibility and soluble interleukin-2 receptor production. PLoS Genet. 2009; 5:e1000322. 10.1371/journal.pgen.1000322 19119414 PMC2602853

[32] Laurent C, Dorothée G, Hunot S, Martin E, Monnet Y, Duchamp M, Dong Y, Légeron FP, Leboucher A, Burnouf S, Faivre E, Carvalho K, Caillierez R, et al. Hippocampal T cell infiltration promotes neuroinflammation and cognitive decline in a mouse model of tauopathy. Brain. 2017; 140:184–200. 10.1093/brain/aww270 27818384 PMC5382942

[33] Oh J, Lee HJ, Song JH, Park SI, Kim H. Plasminogen activator inhibitor-1 as an early potential diagnostic marker for Alzheimer’s disease. Exp Gerontol. 2014; 60:87–91. 10.1016/j.exger.2014.10.004 25304332

[34] Akhter H, Huang WT, van Groen T, Kuo HC, Miyata T, Liu RM. A Small Molecule Inhibitor of Plasminogen Activator Inhibitor-1 Reduces Brain Amyloid-β Load and Improves Memory in an Animal Model of Alzheimer’s Disease. J Alzheimers Dis. 2018; 64:447–57. 10.3233/JAD-180241 29914038

[35] Marksteiner J, Imarhiagbe D, Defrancesco M, Deisenhammer EA, Kemmler G, Humpel C. Analysis of 27 vascular-related proteins reveals that NT-proBNP is a potential biomarker for Alzheimer’s disease and mild cognitive impairment: a pilot-study. Exp Gerontol. 2014; 50:114–21. 10.1016/j.exger.2013.12.001 24333505 PMC4312837

[36] Wang J, Yuan Y, Cai R, Huang R, Tian S, Lin H, Guo D, Wang S. Association between Plasma Levels of PAI-1, tPA/PAI-1 Molar Ratio, and Mild Cognitive Impairment in Chinese Patients with Type 2 Diabetes Mellitus. J Alzheimers Dis. 2018; 63:835–45. 10.3233/JAD-171038 29689724

[37] Trollor JN, Smith E, Baune BT, Kochan NA, Campbell L, Samaras K, Crawford J, Brodaty H, Sachdev P. Systemic inflammation is associated with MCI and its subtypes: the Sydney Memory and Aging Study. Dement Geriatr Cogn Disord. 2010; 30:569–78. 10.1159/000322092 21252552

[38] Shpakov AO, Granstrem AO. C-peptide structure, functions and molecular mechanisms of action. Tsitologiia. 2013; 55:16–27. 23662575

[39] Shpakov AO, Granstrem OK. C-peptide physiological effects. Ross Fiziol Zh Im I M Sechenova. 2013; 99:196–211. 23650733

[40] Chapouly C, Tadesse Argaw A, Horng S, Castro K, Zhang J, Asp L, Loo H, Laitman BM, Mariani JN, Straus Farber R, Zaslavsky E, Nudelman G, Raine CS, John GR. Astrocytic TYMP and VEGFA drive blood-brain barrier opening in inflammatory central nervous system lesions. Brain. 2015; 138:1548–67. 10.1093/brain/awv077 25805644 PMC4614128

[41] Argaw AT, Gurfein BT, Zhang Y, Zameer A, John GR. VEGF-mediated disruption of endothelial CLN-5 promotes blood-brain barrier breakdown. Proc Natl Acad Sci USA. 2009; 106:1977–82. 10.1073/pnas.0808698106 19174516 PMC2644149

[42] Odent Grigorescu G, Rosca AM, Preda MB, Tutuianu R, Simionescu M, Burlacu A. Synergic effects of VEGF-A and SDF-1 on the angiogenic properties of endothelial progenitor cells. J Tissue Eng Regen Med. 2017; 11:3241–52. 10.1002/term.2233 27943613

[43] Cho SJ, Park MH, Han C, Yoon K, Koh YH. VEGFR2 alteration in Alzheimer’s disease. Sci Rep. 2017; 7:17713. 10.1038/s41598-017-18042-1 29255164 PMC5735090

[44] Ciesielska N, Sokołowski R, Mazur E, Podhorecka M, Polak-Szabela A, Kędziora-Kornatowska K. Is the Montreal Cognitive Assessment (MoCA) test better suited than the Mini-Mental State Examination (MMSE) in mild cognitive impairment (MCI) detection among people aged over 60? Meta-analysis. Psychiatr Pol. 2016; 50:1039–52. 10.12740/PP/45368 27992895

[45] Nasreddine ZS, Phillips NA, Bédirian V, Charbonneau S, Whitehead V, Collin I, Cummings JL, Chertkow H. The Montreal Cognitive Assessment, MoCA: a brief screening tool for mild cognitive impairment. J Am Geriatr Soc. 2005; 53:695–99. 10.1111/j.1532-5415.2005.53221.x 15817019

[46] Petersen RC. Mild cognitive impairment as a diagnostic entity. J Intern Med. 2004; 256:183–94. 10.1111/j.1365-2796.2004.01388.x 15324362

[47] Winblad B, Palmer K, Kivipelto M, Jelic V, Fratiglioni L, Wahlund LO, Nordberg A, Bäckman L, Albert M, Almkvist O, Arai H, Basun H, Blennow K, et al. Mild cognitive impairment--beyond controversies, towards a consensus: report of the International Working Group on Mild Cognitive Impairment. J Intern Med. 2004; 256:240–46. 10.1111/j.1365-2796.2004.01380.x 15324367

