# Supplementary material for: Identification of inflammatory and vascular markers associated with mild cognitive impairment
Source: Aging (Albany NY). 2019 Apr 30;11(8):2403–19. doi: 10.18632/aging.101924 (PMC6520012; doi:10.18632/aging.101924)
Supplement: Supplementary Tables [file aging-11-101924-s001.pdf]

SUPPLEMENTARY TABLES

Supplementary Table 1. Relationship of plasma markers with cognitive rerformances in different groups.

Markers	MCI + Control				MCI				Control			
	unadjusted		adjusted		unadjusted		adjusted		unadjusted		adjusted	
	ρ	p value	r	p value	ρ	p value	r	p value	ρ	p value	r	p value
TNF-α												
MMSE	-0.098	0.343	-0.132	0.221	0.056	0.704	0.069	0.659	0.185	0.214	0.028	0.864
MoCA	-0.047	0.654	-0.060	0.579	-0.047	0.752	-0.019	0.902	0.273	0.069	0.197	0.216
sTNFR 1												
MMSE	-0.121	0.208	-0.198	**0.045**	-0.123	0.367	-0.269	0.056	-0.013	0.928	0.011	0.940
MoCA	-0.235	**0.015**	-0.230	**0.020**	-0.314	**0.019**	-0.357	**0.010**	-0.058	0.681	0.085	0.567
sTNFR 2												
MMSE	-0.153	0.289	-0.157	0.302	-0.321	0.136	0.268	0.072	0.032	0.875	-0.134	0.542
MoCA	-0.119	0.417	-0.126	0.411	-0.105	0.642	-0.105	0.679	0.028	0.890	-0.160	0.465
hsIL-6												
MMSE	0.083	0.433	0.195	0.073	0.119	0.404	0.268	0.072	-0.019	0.905	0.090	0.607
MoCA	0.014	0.898	0.005	0.962	-0.049	0.738	-0.168	0.266	0.051	0.757	0.141	0.418
sgp130												
MMSE	-0.079	0.418	-0.030	0.762	0.153	0.266	0.124	0.389	-0.164	0.239	-0.150	0.313
MoCA	-0.075	0.446	-0.055	0.585	0.093	0.503	0.056	0.697	-0.172	0.228	-0.099	0.507
IP-10												
MMSE	-0.182	0.057	0.009	0.928	-0.074	0.589	0.083	0.564	-0.138	0.319	-0.049	0.743
MoCA	-0.160	0.100	0.028	0.781	0.001	0.995	0.101	0.482	-0.053	0.708	0.057	0.700
CXCL13												
MMSE	-0.027	0.780	0.033	0.739	-0.017	0.902	-0.071	0.618	-0.175	0.205	-0.084	0.569
MoCA	-0.119	0.223	0.021	0.835	-0.076	0.583	-0.028	0.844	-0.182	0.198	-0.138	0.348
sIL-2Rα												
MMSE	-0.207	**0.030**	-0.169	0.089	-0.269	**0.045**	-0.201	0.158	0.000	0.999	-0.043	0.774
MoCA	-0.305	**0.001**	-0.244	**0.013**	-0.464	**0.000**	-0.420	**0.002**	-0.011	0.904	0.050	0.735

6Ckine												
MMSE	-0.138	0.150	-0.159	0.108	-0.130	0.340	-0.282	**0.045**	0.022	0.877	0.127	0.390
MoCA	-0.104	0.285	-0.039	0.694	-0.198	0.147	-0.208	0.143	0.172	0.223	0.258	0.077
CTACK												
MMSE	-0.064	0.507	-0.120	0.227	0.064	0.639	-0.152	0.285	-0.005	0.972	-0.015	0.921
MoCA	-0.076	0.436	-0.007	0.941	0.061	0.656	0.058	0.684	-0.090	0.526	-0.094	0.527
hsCRP												
MMSE	0.057	0.556	0.025	0.802	0.036	0.795	-0.018	0.902	-0.045	0.751	0.023	0.878
MoCA	0.037	0.708	0.045	0.656	-0.094	0.500	-0.036	0.803	0.031	0.831	0.219	0.139
IL-8												
MMSE	0.111	0.266	0.176	0.089	-0.061	0.665	0.110	0.458	0.172	0.238	0.242	0.118
MoCA	0.182	0.072	0.181	0.080	0.021	0.884	0.011	0.940	0.298	**0.042**	0.261	0.091
C-peptide												
MMSE	-0.174	0.073	-0.161	0.110	-0.102	0.459	-0.168	0.243	0.188	0.182	0.210	0.161
MoCA	-0.130	0.188	-0.080	0.429	-0.112	0.418	-0.231	0.107	0.282	**0.047**	0.389	**0.008**
MCP-1												
MMSE	0.048	0.625	0.100	0.323	-0.043	0.754	0.034	0.814	0.064	0.654	0.102	0.501
MoCA	0.104	0.292	0.139	0.168	0.140	0.314	0.139	0.335	0.095	0.512	0.096	0.527
VEGFA												
MMSE	0.174	0.071	0.157	0.117	-0.055	0.690	-0.048	0.739	0.115	0.414	0.107	0.474
MoCA	0.155	0.115	0.134	0.181	-0.064	0.647	-0.216	0.133	0.185	0.195	0.224	0.131
IL-4												
MMSE	0.076	0.482	0.145	0.192	0.007	0.964	0.031	0.850	-0.048	0.756	0.165	0.315
MoCA	0.146	0.179	0.181	0.103	0.123	0.431	0.110	0.504	0.022	0.888	0.169	0.305
Leptin												
MMSE	-0.069	0.498	-0.082	0.440	-0.235	0.097	-0.263	0.077	-0.049	0.733	0.041	0.793
MoCA	-0.014	0.897	-0.022	0.834	-0.194	0.176	-0.223	0.137	-0.008	0.959	-0.140	0.366

PAI-1												
MMSE	0.130	0.187	0.074	0.474	-0.132	0.346	-0.143	0.332	-0.037	0.797	0.041	0.788
MoCA	0.140	0.162	0.014	0.891	-0.106	0.457	-0.139	0.347	0.067	0.647	-0.078	0.610
NTproBNP												
MMSE	0.100	0.389	0.132	0.278	-0.081	0.610	0.065	0.703	-0.082	0.638	-0.050	0.797
MoCA	0.076	0.520	0.036	0.768	0.146	0.362	0.123	0.469	-0.303	0.087	-0.481	**0.008**
TIMP-2												
MMSE	-0.103	0.321	-0.117	0.279	-0.027	0.856	-0.206	0.185	-0.225	0.129	-0.064	0.691
MoCA	-0.179	0.088	-0.196	0.068	-0.090	0.548	-0.200	0.199	-0.286	0.057	-0.280	0.076
T4 Total												
MMSE	-0.072	0.496	-0.058	0.593	-0.007	0.961	-0.025	0.869	0.034	0.831	-0.079	0.652
MoCA	-0.050	0.642	-0.165	0.130	-0.029	0.842	-0.201	0.176	0.034	0.837	-0.125	0.475

Note: MCI + Control = all participants included in the study; MCI = patients with mild cognitive impairment; Control = cognitively healthy controls; MMSE, Mini-Mental State Exam; MoCA, Montreal Cognitive Assessment; unadjusted, ρ and p value were obtained from the Spearman correlation analysis; adjusted, r and p value were obtained from the Partial correlation analysis after adjustment for age, gender proportion, education level, GDS and GAI scores. Significance (bold values), $p < 0.05$.

Supplementary Table 2. Assessment results of the risk of having cognitive impairment in a simple linear regression model across different groups.

Markers	MCI + Control		MCI		Control	
	B	p value	B	p value	B	p value
TNF-α						
MMSE	-3.512	0.296	1.313	0.769	1.015	0.423
MoCA	-3.456	0.491	-2.002	0.762	7.043	0.137
sTNFR 1						
MMSE	-4.016	**0.040**	-4.114	0.110	-0.012	0.988
MoCA	-8.329	**0.005**	-10.527	**0.009**	-0.799	0.770
sTNFR 2						
MMSE	-1.276	0.648	-1.415	0.678	-0.668	0.648
MoCA	-1.685	0.677	-1.212	0.808	-0.734	0.880

hsIL-6						
MMSE	0.751	0.443	0.643	0.631	-0.005	0.989
MoCA	-0.415	0.783	-1.849	0.379	0.494	0.703
sgp130						
MMSE	-1.952	0.453	12.163	0.100	-0.692	0.340
MoCA	-3.713	0.348	10.721	0.362	-1.847	0.472
IP-10						
MMSE	-1.775	0.083	-0.314	0.814	-0.398	0.350
MoCA	-2.026	0.200	0.151	0.942	-0.506	0.743
CXCL13						
MMSE	-0.027	0.988	-0.183	0.938	-0.428	0.520
MoCA	-1.172	0.664	-0.426	0.909	-2.526	0.282
sIL-2Rα						
MMSE	-4.526	**0.013**	-5.035	0.057	-0.330	0.619
MoCA	-9.713	**0.000**	-14.545	**0.000**	-1.027	0.662
6Ckine						
MMSE	-4.708	0.079	-6.920	0.106	0.601	0.501
MoCA	-3.823	0.357	-10.412	0.120	4.682	0.141
CTACK						
MMSE	-1.088	0.491	0.121	0.959	0.183	0.749
MoCA	-0.979	0.685	1.586	0.650	-0.666	0.743
hsCRP						
MMSE	0.184	0.651	0.132	0.806	-0.072	0.641
MoCA	0.076	0.904	-0.312	0.713	0.302	0.593
IL-8						
MMSE	0.912	0.373	-0.808	0.590	0.460	0.170
MoCA	2.439	0.121	-0.398	0.864	2.260	0.082
C-peptide						
MMSE	-1.759	**0.015**	-1.348	0.237	0.305	0.235

MoCA	-2.010	0.072	-2.805	0.117	1.847	**0.045**
MCP-1						
MMSE	0.842	0.596	-2.152	0.334	0.607	0.291
MoCA	3.238	0.211	2.942	0.472	0.878	0.668
VEGFA						
MMSE	0.947	0.296	-1.157	0.400	0.223	0.480
MoCA	1.237	0.372	-2.859	0.191	1.134	0.312
IL-4						
MMSE	0.883	0.510	-1.728	0.387	0.444	0.349
MoCA	2.162	0.278	-0.387	0.903	1.415	0.424
Leptin						
MMSE	-0.654	0.327	-1.272	0.137	-0.112	0.677
MoCA	-0.533	0.613	-2.041	0.144	1.062	0.284
PAI-1						
MMSE	0.852	0.337	-1.403	0.261	0.229	0.856
MoCA	1.373	0.325	-1.811	0.366	0.017	0.961
NTproBNP						
MMSE	0.662	0.552	0.471	0.789	-0.207	0.602
MoCA	0.856	0.628	2.643	0.353	-2.133	0.108
TIMP-2						
MMSE	-1.205	0.447	-0.846	0.723	-0.518	0.352
MoCA	-3.442	0.144	-2.062	0.571	-3.762	0.053
T4 Total						
MMSE	0.375	0.590	-0.128	0.970	-0.171	0.365
MoCA	0.071	0.946	-5.782	0.279	-0.368	0.578

Note: Dependent variable: MMSE or MoCA scores; B, regression coefficients; SE, standard error; MCI + Control = all participants included in the study; MCI = patients with mild cognitive impairment; Control = cognitively healthy controls; MMSE, Mini-Mental State Exam; MoCA, Montreal Cognitive Assessment. Significance (bold values), $p < 0.05$.
